# Interventions for wound healing among diabetic patients infected with Staphylococcus aureus: a systematic review

**DOI:** 10.1590/S1516-31802011000300007

**Published:** 2011-05-05

**Authors:** Anacássia Fonseca Lima, Lívia Bandeira Costa, Joás Lucas da Silva, Maria Bernadete Sousa Maia, Eulália Camelo Pessoa Azevedo Ximenes

**Affiliations:** I BSc. Biomedical Scientist, Microorganism Physiology and Biochemistry Laboratory, Universidade Federal de Pernambuco (UFPE), Recife, Pernambuco, Brazil.; II BSc. Biomedical Scientist, Cell Culture Laboratory, Department of Histology and Embryology, Universidade Federal de Pernambuco (UFPE), Recife, Pernambuco, Brazil.; III MSc. Biomedical Scientist, Bioactive Product Biotechnology Laboratory, Universidade Federal de Pernambuco (UFPE), Recife, Pernambuco, Brazil.; IV PhD. Associate Professor, Bioactive Product Pharmacology Laboratory, Universidade Federal de Pernambuco (UFPE), Recife, Pernambuco, Brazil.; V PhD. Associate Professor, Microorganism Physiology and Biochemistry Laboratory, Universidade Federal de Pernambuco (UFPE), Recife, Pernambuco, Brazil.

**Keywords:** Wound healing, Infection, Staphylococcus aureus, Diabetic foot, Leg ulcer, Foot ulcer, Ulcer, Cicatrização de feridas, Infecção, Staphylococcus aureus, Pé diabético, Úlcera da perna, Úlcera do pé, Úlcera

## Abstract

**CONTEXT AND OBJECTIVE:**

*Staphylococcus aureus* is the most frequent agent isolated in diabetic foot infections and may be associated with changes to wound healing times. The aim of this study was to perform a systematic review of the literature, including studies that assessed the efficacy of any clinical or surgical intervention, as well as oral or topical therapy for diabetic ulcers infected with *S. aureus*.

**DESIGN AND SETTING:**

Systematic review with a search conducted in databases.

**METHODS:**

We conducted a systematic review with a comprehensive search in the Lilacs, SciELO, PubMed/Medline, Old Medline, Embase and Cochrane Library databases, for articles published from 1966 to 2010. The articles selected were limited to studies on diabetic patients with wounds infected with *S. aureus* for whom their healing was followed up, with the use of either antibiotics or experimental treatments. Animal studies and those that did not report the wound healing, as well as review articles, were excluded.

**RESULTS:**

Five studies that met the inclusion and exclusion criteria were analyzed.

**CONCLUSIONS:**

There are few studies reporting the healing of wounds infected with *S. aureus* in diabetic patients, although this is the most commonly found pathogen in this type of wound and it frequently consists of methicillin-resistant *S. aureus* (MRSA). There is insufficient evidence to support early use of broad-spectrum antibiotics against MRSA to promote healing of diabetic ulcers, since antibiotic resistance may develop from such treatment. This highlights the need for further studies on the subject.

## RESUMO

**CONTEXTO:**

*Staphylococcus aureus* é o agente mais frequentemente isolado nas infecções de pé em pacientes diabéticos e pode estar associado a mudança no tempo de cicatrização de feridas. O objetivo deste estudo foi realizar uma revisão sistemática da literatura, incluindo estudos que avaliaram a eficácia de qualquer intervenção clínica, cirúrgica, bem como terapia oral ou tópica para o tratamento de úlceras diabéticas infectadas com o *S. aureus*.

**TIPO DE ESTUDO E LOCAL:**

Revisão sistemática com busca realizada em bancos de dados.

**MÉTODOS:**

Realizamos uma revisão sistemática com uma busca abrangente nos bancos de dados Lilacs, SciELO, PubMed/Medline, Old Medline, Embase e no banco de dados da biblioteca Cochrane, publicados entre 1966 e 2010. Os artigos selecionados foram limitados aos estudos com feridas infectadas por *S. aureus* de pacientes diabéticos, que tiveram cicatrização relatada, quer pela utilização de antibióticos ou por substâncias experimentais. Foram excluídos os estudos com animais e os que não relataram a cicatrização das feridas, bem como artigos de revisão.

**RESULTADOS:**

Foram analisados cinco estudos que obedeceram aos critérios de inclusão e exclusão.

**CONCLUSÕES:**

Raros estudos relataram cicatrização de feridas infectadas com *S. aureus* em pacientes diabéticos, embora este seja o patógeno mais comumente encontrado neste tipo de ferida, sendo frequentemente resistente à meticilina MRSA (*methicillin-resistant S. aureus*). Não há evidências suficientes que suportem a utilização precoce de antibióticos de amplo espectro contra MRSA para promoção da cicatrização de úlceras diabéticas, uma vez que o desenvolvimento de resistência a antibióticos pode decorrer desse tipo de tratamento. Isso evidencia a necessidade de novos estudos sobre o assunto.

## INTRODUCTION

Ischemia, neuropathy and infection are the three pathological components that lead to diabetic foot complications, and they frequently occur together as an etiological trio. Infection of foot ulcers is commonly seen in diabetic patients and is a substantial morbid event.^1^ *Staphylococcus aureus* (*S. aureus*) is, by far, the most frequent pathogen isolated in diabetic foot infections, either singly or as a component of mixed infection.^2^

Diabetes also causes structural and functional changes within the arteriolar and capillary systems, notably with thickening of the basement membrane.^3^ This thickened membrane impairs leukocytes migration and hampers the normal hyperemic or vasodilatory response to injury, thus simultaneously increasing the susceptibility to injury while also blunting the typical manifestations of such an injury.^4^ Because of this blunted neuroinflammatory response, diabetic patients lack a crucial component of the body's natural first line of defense against pathogens and thus are more susceptible to an ensuing foot infection.^5^ The present study will provide knowledge of interventions that lead to healing of wounds infected with *S. aureus*.

## OBJECTIVE

The aim of this study was to perform a systematic review of the scientific literature, including studies that assessed the efficacy of any clinical or surgical intervention, as well as oral or topical therapy for diabetic ulcers infected with *S. aureus*.

## METHODS

Searches to locate articles relating to the healing of wounds infected with *S. aureus* in diabetic patients were conducted in the Medline/PubMed (Medical Literature Analysis and Retrieval System Online), Lilacs (Literatura Latino-Americana e Caribe em Ciências da Saúde), SciELO (Scientific Electronic Library Online), Old Medline, Embase (Excerpta Medica) and Cochrane Library databases. Searches were restricted to the period from 1966 to 2010.

The databases were searched using a comprehensive strategy (Table 1), along with MeSH (Medical Subject Headings) and text words, including the following exhaustive list of synonyms: wound healing, infection, *S. aureus*, diabetic foot, leg ulcer, foot ulcer and ulcer. Bibliographic references in relevant review articles were also examined for eligible trials. In addition, thesis databases were searched manually, references of references were searched, specialists were consulted and contacts were made with the pharmaceutical industry. Searches were also carried out in Clinical Trials.gov and in the Current Controlled Trials. References, and any relevant studies identified were scrutinized for additional citations.

**Table 1.** Search strategy

Database	Date	Search filters
Embase/Lilacs/SciELO/Cochrane	06/09/10	Wound [Text Word] AND Diabetes [Text Word] AND Staphylococcus aureus [MeSH]
Embase/Lilacs/SciELO/Cochrane	13/09/10	Wound healing [MeSH] AND Diabetes [Text Word] AND Staphylococcus aureus [MeSH]
Embase/Lilacs/SciELO/Cochrane	17/09/10	Wound healing [MeSH] AND Diabetic foot [MeSH] AND Wound [Text Word] AND Leg Ulcer [MeSH] AND Foot Ulcer [MeSH] AND Ulcer [MeSH] AND Diabetes [Text Word] AND Staphylococcus aureus [MeSH]
Medline/PubMed	19/09/10	("wound healing"[MeSH Terms] OR ("wound"[All Fields] AND "healing"[All Fields]) OR "wound healing"[All Fields]) AND ("diabetic foot"[MeSH Terms] OR ("diabetic"[All Fields] AND "foot"[All Fields]) OR "diabetic foot"[All Fields]) AND ("wounds and injuries"[MeSH Terms] OR ("wounds"[All Fields] AND "injuries"[All Fields]) OR "wounds and injuries"[All Fields] OR "wound"[All Fields]) AND ("leg ulcer"[MeSH Terms] OR ("leg"[All Fields] AND "ulcer"[All Fields]) OR "leg ulcer"[All Fields]) AND ("foot ulcer"[MeSH Terms] OR ("foot"[All Fields] AND "ulcer"[All Fields]) OR "foot ulcer"[All Fields]) AND ("ulcer"[MeSH Terms] OR "ulcer"[All Fields]) AND ("diabetes mellitus"[MeSH Terms] OR ("diabetes"[All Fields] AND "mellitus"[All Fields]) OR "diabetes mellitus"[All Fields] OR "diabetes"[All Fields] OR "diabetes insipidus"[MeSH Terms] OR ("diabetes"[All Fields] AND "insipidus"[All Fields]) OR "diabetes insipidus"[All Fields]) AND ("staphylococcus aureus"[MeSH Terms] OR ("staphylococcus"[All Fields] AND "aureus"[All Fields]) OR "staphylococcus aureus"[All Fields]) AND ("humans"[MeSH Terms])

Trial selection, data abstraction and data synthesis were performed by two authors independently. Disagreements were solved by discussion.

The articles selected were limited to studies on diabetic patients with wounds infected with *S. aureus* that were treated for infection using any clinical or surgical intervention, as well as oral or topical therapy for diabetic ulcers infected with *S. aureus*. We excluded animal studies, studies on non-diabetic patients, studies that did not report wound healing and literature review articles.

## RESULTS

**Figure 1.**
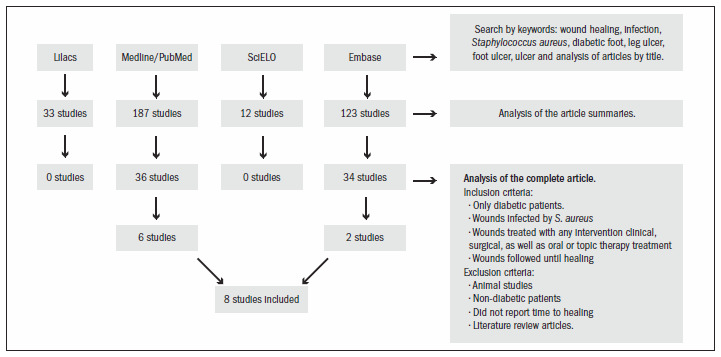
Methodological flow of search strategy.

Among all the articles initially identified through the electronic search, six items from the Medline/PubMed database and two from Embase relating to the healing of wounds infected with *Staphylococcus aureus* in diabetic patients published between 1999 and 2010 were fully recovered for further evaluation. In cases of repeated studies, only one search source was taken into consideration. There were no randomized clinical trials on this subject. All the studies were observational in nature. The search strategy is shown in Figure 1. This systematic review included a total of eight studies.

In Table 2, the studies have been displayed in chronological order emphasizing the type of study, number of patients used in each study, treatment administered to the wounds, healing time and conclusion obtained.

**Table 2.** Analysis of selected studies

Author (year)	Type of study	Sample	Staphylococcus aureus Wound Infection (n)	Treatment	Healing time (mean)	Conclusion
Tentolouris et al.^6^	Prevalence	75 diabetic patients	MSSA (18) MSRA (12)	Antibiotic therapy (clindamycin or amoxicillin/clavulanic acid)	17.8 weeks (MSSA) 35.4 weeks (MRSA)	Methicillin-resistant S. aureus (MRSA) infection is common in diabetic foot ulcers and is associated with previous antibiotic treatment and prolonged time to healing.
Dang et al.^7^	Prevalence	63 diabetic patients	MSSA (26) MSRA (19)	Antibiotic therapy (clindamycin and amoxicillin/clavulanic acid)	12 weeks (all ulcers of the study)	MRSA infection is associated with slower ulcer healing and is likely to have impact on the treatment cost. The problem of MRSA continues to increase despite precautions taken to prevent MRSA spread. There is a need for a multi-center study looking into the prevalence of MRSA in diabetic foot ulcer and how this can be reduced in the diabetic foot clinic.
Hartemann-Heurtier et al.^8^	Longitudinal	180 diabetic patients	MSSA (64) MSRA (29)	Antibiotic therapy (amoxicillin/clavulanic acid and aminoglycoside)	4.6 ± 8.4 months (MDRO+) 6.7 ± 13 months (MDRO–)	About one-third of patients with a history of previous hospitalization for the same wound and 25% patients with osteomyelitis had MDRO-positive specimens. Positive MDRO status is not associated with a longer time to healing.
Cavallini^9^	Longitudinal	10 diabetic patients	Staphylococcus aureus (7)	Surgical debridement, antibiotic therapy and dermal graft with meshes of autologous fibroblasts	7 patients healed at 14.2 weeks	Autologous ﬁbroblast grafts should be considered to be a correct approach for treating chronic and complicated deep ulcers, given that when accompanied by antibiotic therapy, wound healing is optimized.
Kim et al.^10^	Longitudinal	52 diabetic patients	MSSA (16) MSRA (7)	Modiﬁed resection arthroplasty	25.6 ± 6.2 days	Modiﬁed resection arthroplasty for toe deformities with chronic infected ulcers in diabetic patients is a good treatment alternative to toe amputation.
Richard et al.^11^	Longitudinal	188 diabetic patients	MSSA (62) MSRA (37)	Antibiotic therapy (fluoroquinolone, rifampicin and clindamycin)	14 weeks (MDRO+) 10 weeks (MDRO–)	MDRO presence seems to have no significant impact on healing time if early aggressive treatment is adopted, including empirical broad-spectrum antibiotic treatment, later adjusted according to microbiological findings.
ElMakki Ahmed et al.^12^	Prospective cohort	122 diabetic patients	Staphylococcus aureus (56)	Surgical debridement and. antibiotic therapy (amoxicillin/clavulanic acid)	16 ± 8 weeks	Outcome from diabetic foot is dependent on degree of limb ischemia, size of the ulcer, and severity of sepsis, and it can be optimized through debridement and removal of infected bone fragments, in conjunction with antibiotic therapy.
Nagoba et al.^13^	Retrospective	115 diabetic patients	Staphylococcus aureus (47)	3% citric acid gel daily	106 cases healed with 16-34 applications	These results indicate that citric acid treatment is highly effective for controlling the infecting organism, which is paramount to the success of healing.

n = number of infected wounds; MSSA = methicillin-sensitive Staphylococcus aureus; MRSA = methicillin-resistant Staphylococcus aureus; MDRO = multidrug resistant microorganisms.

## DISCUSSION

Few studies have reported the healing of wounds infected with *S. aureus* in diabetic patients. The eight studies examined here were all conducted over a similar time span. The first study was performed in 1999;^6^ the second was a continuation of the first, in 2003;^7^ the third was in 2004;^8^ the fourth was in 2007;^9^ another two articles appeared in 2008;^10,11^ and the last and most recent two appeared in 2010.^12,13^ This small number of studies may be due to difficulty in monitoring diabetic patients from the onset of the infection until wound healing, thus making it complex to obtain a homogeneous sample. It is also due to difficulties in obtaining a diagnosis of infection. It can be very difficult to define the infection, especially in the presence of peripheral ischemia, and there are no clear criteria available for distinguishing infection from non-pathogenic colonization.^14^

The samples in the studies analyzed were of significant size, with numbers of patients with wounds exceeding 50, with the exception of one study.^9^ This study evaluated wound healing in 10 diabetic patients, among whom seven were infected with *S. aureus*. The small sample in that study can be explained by the experimental features of the treatment used. Dermal grafts were used, with meshes of autologous fibroblasts. This treatment proved to be very promising and the authors encouraged further studies with a higher number of patients.

Another article reported on 30 wounds infected with *S. aureus* among 75 patients studied: 18 of these were methicillin-sensitive *S. aureus* (MSSA) and 12 were methicillin-resistant *S. aureus* (MRSA).^6^ Dang et al.^7^ reported on wound development in 63 patients with diabetes, of whom 26 had MSSA and 19 had MRSA. Hartemann-Heurtier et al.^8^ studied 180 patients who developed wounds: 64 colonized by MSSA and 29 by MRSA. Cavallini^9^ did not report on the susceptibility of *S. aureus* to methicillin. Kim et al.^10^ used modiﬁed resection arthroplasty to completely remove the infected phalangeal bone and to suture the dorsal wound and extensor tendon of 52 diabetic patients: 23 of these were infected by *S. aureus*, including seven cases of MRSA. Richard et al.^11^ followed up 188 diabetic patients, among whom 62 had wounds infected with MSSA and 37 with MRSA. In a prospective cohort study, ElMakki Ahmed et al.^12^ assessed the risk factors associated with hallux ulceration and the incidence of healing or amputation in 122 diabetic patients, of whom 56 were infected by *S. aureus*. Nagoba et al.^13^ investigated susceptibility to citric acid *in vitro* and in ulcers of different Wagner grades infected with a variety of bacteria (47 with *S. aureus*) from 115 diabetic patients. The Wagner grade is determined based on the depth of the skin lesion and the presence or absence of infection and gangrene, and is divided into six grades ranging from grade zero to grade five.^15^ The last two authors did not report the susceptibility of *S. aureus* to methicillin.

All the authors found that *S. aureus* was the most frequent microorganism infecting wounds in diabetic patients, but there was disagreement over whether the presence of the organism influenced the healing time. The high prevalence may be due to the fact that this microorganism is a skin colonizer that becomes opportunistic in immunocompromised people such as diabetic patients. The large number of wounds infected with MRSA can be correlated with previous use of broad-spectrum antibiotics.^16^

In the articles selected, the antibiotic treatments used were similar: clindamycin and amoxicillin/clavulanic acid were used in most of the studies. Tentolouris et al.^6^ and Dang et al.^7^ stated that specific antibiotic therapy for MRSA encouraged microbial resistance and was unnecessary since, according to these authors, MRSA could be eradicated by means of regular debridement, topical treatments and isolation in the foot clinic without the requirement for treatment with specific antibiotics (Table 2). However, MRSA was associated with a longer time for healing. ElMakki Ahmed et al.^12^ did not report whether there was any association between healing time and susceptibility to infecting microorganisms. Nonetheless, even though these authors advocated that medical therapy alone was the most effective method, they stated that surgical debridement with removal of all the infected area was essential for wound healing.

Hartemann-Heurtier et al.,^8^ Richard et al.^11^ and ElMakki Ahmed et al.^12^ administered similar antibiotic therapies. All of them started their patients on broad-spectrum treatment for infections, and the antibiotic therapy was adapted based on the results from microbiological studies, so that it would cover the most likely pathogenic organisms. Hartemann-Heurtier et al.^8^ agreed that indiscriminate use of broad-spectrum antibiotics promoted the emergence of resistance, but they argued that MRSA was acquired more often from cross-transmission than from antibiotic overuse. According to Richard et al.,^11^ isolation of multidrug resistant microorganisms (MDRO) seemed to have no significant impact on healing time when early aggressive treatment of wound infection, including immediate broad-spectrum antibiotics (active against MRSA) were administered, followed by adjustment according to culture results.

With regard to healing time, Tentolouris et al.^6^ and Dang et al.^7^ reported significant differences between ulcers infected with MRSA and with MSSA. The healing times observed by the first authors were 17.8 weeks (MSSA) and 35.4 weeks (MRSA), while the second authors reported a healing time of 12 weeks, regardless of the infecting pathogen. Hartemann-Heurtier et al.^8^ and Richard et al.^11^ ranked MRSA among MDRO. Hartemann-Heurtier et al.^8^ found healing times of 4.6 ± 8.4 months (MDRO+) and 6.7 ± 13 months (MDRO–), and Richard et al.^11^ found 14 weeks (MDRO+) and 10 weeks (MDRO–). ElMakki Ahmed et al.^12^ did not report whether there was any association between healing time and susceptibility to infecting microorganisms, and the mean healing time was 16 ± 8 weeks for all wounds in the study.

Kim et al.^10^ reported that no antibiotics were used in conjunction with the surgical technique, but showed that with the use of modiﬁed resection arthroplasty, it was possible to salvage most of the toes with infected wounds and avoid amputation, thereby leading to a mean healing time of 25.6 ± 6.2 days. Nagoba et al.^13^ observed that citric acid treatment promoted healing after 16-34 applications, and that this was highly effective for controlling infections and for successfully managing diabetic foot ulcers without deep osteomyelitis.

Among the eight studies analyzed in this review, two were related to the presence of methicillin-resistant *Staphylococcus aureus* (MRSA)^6,7,9^ with longer healing times. Another two articles that were analyzed claimed that there was no relationship between MRSA and increased healing time^8,10^ and four papers did not study this association.^9,10,12,13^ Three articles^7,12^ advocated that surgical debridement with removal of all the infected area was a very appropriate treatment, certainly because ulcers heal more quickly if their surfaces are clean and if sinuses are laid open.^17^

Most of the studies used antibiotic therapy as the priority treatment, except for three. Cavallini^9^ used a combination of antibiotic therapy and debridement with dermal grafts using meshes of autologous fibroblasts, Kim et al.^10^ used the surgical technique of modiﬁed resection arthroplasty and Nagoba et al.^13^ used a treatment with citric acid. These articles show that alternative therapies may be an effective alternative to the indiscriminate use of antibiotics.

The studies that did not observe any relationship between the presence of MRSA and longer healing time had the common feature of the use of early aggressive treatment of wound infection. This feature may explain this finding, and justify the use of this type of treatment, since using specific therapy can enhance the healing of infected wounds. The Brazilian National Sanitary Surveillance Agency (Agência Nacional de Vigilância Sanitária; Anvisa) advocates the use of vancomycin or amoxicillin-clavulanic acid for treating MRSA, since this requires specifically targeted antibiotic therapy.^1^ Moreover, the studies that reported an association between healing time and presence of MRSA showed indiscriminate use of antibiotics and a predisposing factor for increased infection by this pathogen.

We did not find any systematic review that was similar to what has been presented here. We found systematic reviews on diabetic foot, chronic wounds and *S. aureus* colonization with conclusive results,^18-27^ but no reviews reporting the healing of wounds infected with *S. aureus*, in diabetic patients.

There are few studies reporting the healing of wounds infected with *S. aureus* in diabetic patients, although this is the most commonly pathogen found in this type of wound and it sometimes consists of MRSA. Studies that used early and aggressive treatment against MRSA infections reported that such therapy can make the healing time similar to that found in other infections. Surgical debridement with removal of all the infected area was also observed to be an important tool for wound healing. Alternative therapies for wound treatment, such as the use of meshes with dermal grafts of autologous fibroblasts, the surgical technique of modiﬁed resection arthroplasty and treatment with citric acid are promising. We could not find any work reporting the use of natural substances for treating wound infections in diabetic patients. Thus, the possibility arises that there may be opportunities to find widespread naturally occurring substances with antimicrobial activity that could serve as alternative treatments.

Wound infection in diabetic patients is a public health problem. Finding a balance between effective antibiotic therapy and control over promotion of bacterial resistance is a challenge. Alternative treatments that can be used in combination with antibiotic therapy may be a way to solve problems relating to long periods of hospitalization, since a prolonged stay in hospital just contributes towards infection with multidrug-resistant strains, either through inadequate antibiotic therapy or through cross-contamination.

## CONCLUSIONS

There is insufficient evidence to support the use of early and aggressive antimicrobial therapy against MRSA to promote healing of diabetic ulcers, since potentially serious development of antimicrobial resistance can result from such treatment. This highlights the need for a randomized controlled trial on this subject.
